# Assessment of natural groundwater reserve of a morphodynamic system using an information-based model in a part of Ganga basin, Northern India

**DOI:** 10.1038/s41598-022-10254-4

**Published:** 2022-04-13

**Authors:** N. C. Mondal, V. Ajaykumar

**Affiliations:** 1 Earth Process Modeling Group, CSIR-National Geophysical Research Institute, Hyderabad, 500 007 India; 2 Academy of Scientific and Innovative Research (AcSIR), Ghaziabad, 201 002 India

**Keywords:** Sustainability, Hydrology

## Abstract

Assessment of morphodynamic groundwater reserves is important for the sustainable management of water resources. It is a truth that groundwater resource evaluation is anxious with the ambiguity of its several factors and employing methods. Thus, an information-based model has been hypothesized to assess natural groundwater reserves in a morphodynamic system in a part of the Ganga basin of Northern India, where the spatial variability in natural groundwater reserve exists. Marginal information of rainfall data, and transinformation among the rainfall, and monthly depth to groundwater level measurement at 50 wells in a dense monitoring network were used for evaluating natural groundwater reserve. The results indicate that an average recharge rate is about 246 mm/monsoon and or 32.65% of the seasonal rainfall, and its values are well-correlated with the soil infiltration rate. It has been found that the estimated recharge rates are about 54.08, 45.85, 33.77, 32.48, and 32.14% of the seasonal rainfall in an active flood plain, back swamp, natural levees, flood plain, and palaeochannel, respectively. The calculated annual rainfall input to groundwater reserve is found about 127.98 MCM/monsoon rainfall, which could be employed for sustainable management of groundwater resources in the morphodynamic system of the Ganga river basin.

## Introduction

In a morphodynamic system, recharge is one of the significant parameters for groundwater reserve. It changes groundwater storage comprising various processes such as recharge and discharge. Rainfall is the primary source of groundwater recharge, whereas irrigation areas, rivers, ponds, lakes, etc. are the secondary sources of groundwater recharge. Likewise, evapotranspiration, withdrawal, river base flow, etc., are the main sources of discharge^1,2^. In the morphodynamic system of any river basin, rainfall recharge is very essential for groundwater management. Therefore, it is imperative to estimate natural reserves due to the rainfall. There are several techniques for assessing natural groundwater recharge, such as groundwater balance^3^, base flow measurements^4^, zero flux plane^5^, Darcian method^6^, lysimeters^7^, water table fluctuation^8^, cumulative rainfall departure^9^, measurements of temperature^10^, geoelectrical resistivity measurements^11^, neutron logging of moisture profiles^12^, Gravity Recovery and Climate Experiment (GRACE)^13^, stable isotopes of hydrogen and oxygen^14^, chloride balance method, dating by ^14^C^15^, environmental tritium^12,16–18^, RS & GIS techniques^19^, watershed modelling^20^, and groundwater flow models^21,22^. These methods have their own merits, demerits, and limitations^23^. Out of them, the tritium (^3^H) tracer technique (piston flow model)^16^ is very useful to estimate natural groundwater recharge^12,17,18,24–26^. Because this technique is one of the direct methods, and estimates recharge based on the piston flow model. Because the infiltration due to rainfall or irrigation gets into discrete layers by pushing an equal amount of water beneath it, and finally, added to the groundwater system. The main usefulness of this technique is direct for determining the spot value of recharge due to the precipitation. In terms of accuracy, the tracer technique can be considered more reliable than the other methods. But it is a costly affair, and also time taking to process for the data acquisition to estimate recharge, particularly in a basin-scale of the developing countries.

**Figure 1:**
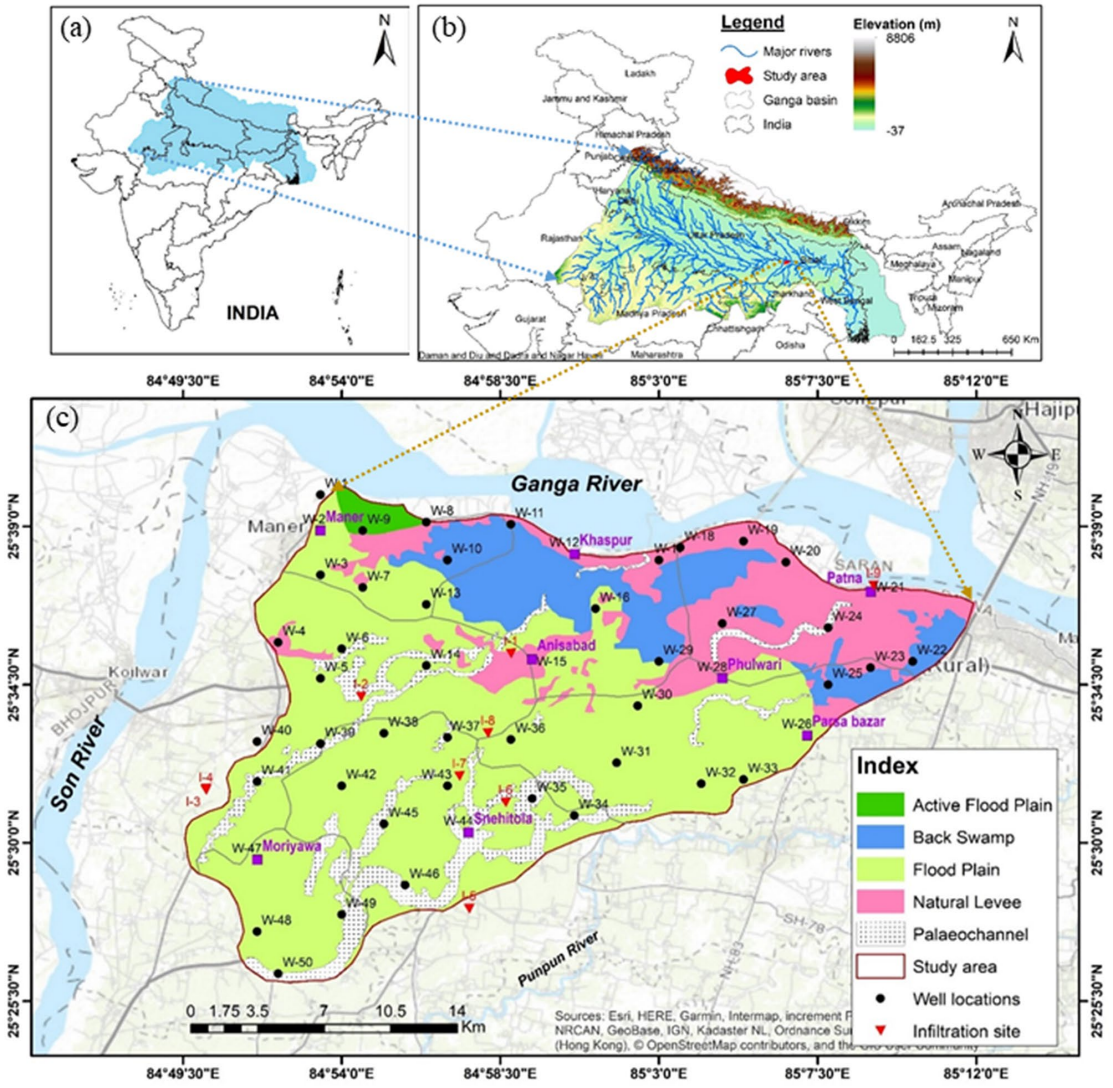
Study area along with the well and infiltration sites on the geomorphic map in a part of the Ganga basin, Northern India (N.C.M.: sketched this figure with the help of ArcGIS Desktop 10.4, http://www.esri.com, CGWB, 201548).

Therefore, it is hypothesized a rapid and straightforward information-based model which can provide a quick and quantitative estimate of natural groundwater reserves. This study explores an information-based statistical method, which has been applied first in the granitic aquifer of Southern India^27^ and then in a basaltic terrain of Central India^28^. Here we used this method to quantify natural recharge in a morphodynamic groundwater system in a part of the Ganga basin, Northern India (Fig. 1). Thus, our objectives of this work are in three folds (1) to assess natural groundwater reserves using the measurement of groundwater level corresponding to rainfall event with the help of an entropy-based model, (2) compare the results with the soil infiltration rate and monsoon groundwater reserve (MGWR) obtained from the water table fluctuation (WTF) method, and (3) also studied its spatial variability in the geomorphological setting.

## Materials and methods

### Description of the study area

The geomorphic system covers an area of 521 km^2^ spreading in the parts of 7-blocks of Patna district, Bihar in Middle Ganga Plain (MGP) in Northern India. The area lies between longitudes: 84°49^/^12^//^ E to 85°13^/^12^//^ E and latitudes:25°25^/^12^//^ N to 25°40^/^’48 ^//^N and falls in the Survey of India Topo-sheets 72G/2 and 72C/14,15. Topography varies from about 45.45 m to 69.00 m, above the mean sea level (amsl) with a general slope from S-W to N-E, and N-directions with minor variations. The mighty River Ganga forms the northern boundary, and other rivers (i.e., Son and Punpun) are draining just outside the area (Fig. 1c). The area has shown five different units as flood plain, active flood plain, natural levee, back swamp, and palaeochannels. The flood plain, basically part of the older flood plain of River Son, is observed throughout the south and western parts^29^. Active flood plain formed due to the activities of Son and Ganga Rivers is confined to the extreme north-western part. But the natural levees form the northern limits of Patna city boundary along the right bank of Holy Ganga river extending 26 km length (east–west direction). The levee is well-exposed along the main Ganga river. The back swamps are observed in the eastern and western parts of the urban area. The palaeochannel (the past Son channel) has also been found at a distance of ~ 35 km west of Patna city, and its orientation is the same as the Son river flow. A thick alluvium sequence exits the pre-Tertiary formations holding a multi-aquifer system. It represents a sedimentary river basin environment with different stages and types of aquifers comprising various grades of sand, silt, and clay, which form the groundwater reservoir due to the reworks of the Son and Ganga rivers. But the rainfall is the main source of groundwater reserve in this study area. It has been found that the maximum rainfall occurs due to the S-W monsoon, and accounts for about 86.2% of total rainfall during 2010–2019. The average normal monsoon rainfall is about 752.3 mm/year (Fig. S1). Types of soil such as sandy loam, sandy clay loam, and clay loam (Fig. S2) are the factors that allow natural groundwater recharge. The sandy loams with clay loam at some places are predominant soil.

### Information theory

Information (entropy) theory was firstly coined by Shannon^30^, who established a quantitative measure of the uncertainty or the information content of a random variable^31^. This information is mainly calculated by the entropy indices such as marginal entropy, joint entropy, and transformation. If it follows for the discrete random variables X = {x_i_}, i = 1, 2, 3…, n, and Y = {y_j_}, j = 1,2, 3,…, m^32,33^, then marginal entropy, H(X) for the variable, X defined as.

1 $$H(X)\, = \,\, - \,\sum\limits_{i\, = \,1}^{n} {\,p(x_{i} )\,\ln \,p(x_{i} )}$$

where, p(x_i_): the probability of *i*th random variable, X = {x_i_}, n: is the number of observations, and the value of total probabilities = $\,\sum\limits_{i\, = \,1}^{n} {\,p(x_{i} )\,} = 1$.

The H(X) of the Eq. (1) provides the amount of information or uncertainty that X has, and similarly, the marginal entropy, H(Y) shows the information or uncertainty that Y has^32^. If both the X and Y variables are stochastically dependent, then the joint entropy, H (X, Y) is expressed as.

2 $$H(X,Y)\, = \,\, - \,\sum\limits_{i\, = \,1}^{n} {\sum\limits_{j = 1}^{m} {p(x_{i} ,y_{j} )} \,\ln \,p(x_{i} ,y_{j} )}$$

In which, p (x_i,_ y_j_) is the joint probability between x_i,_ and y_j._

Transinformation (called mutual information) between two variables describes the amount of information, which is common in both two stochastically dependent variables, X and Y. It is defined as^33^.

3 $$T(X,Y) = \sum\limits_{i = 1}^{n} {\sum\limits_{j = 1}^{m} {p(x_{i} ,y_{j} )\ln \left[ {\frac{{p(x_{i} ,y_{j} )}}{{p(x_{i} )p(y_{j} )}}} \right]} }$$

where, p (x_i_, y_j_): the joint probability of x_i_ and y_j_, and p(x_i_) and p(y_j_): both discrete probability occurrence of x_i_ and y_j_, respectively. The unit of information measure depends on the logarithmic base used. We had used a base ‘2’ in our computation, then the unit is ‘bit’^34^.

### Entropy-based model development

**Figure 2:**
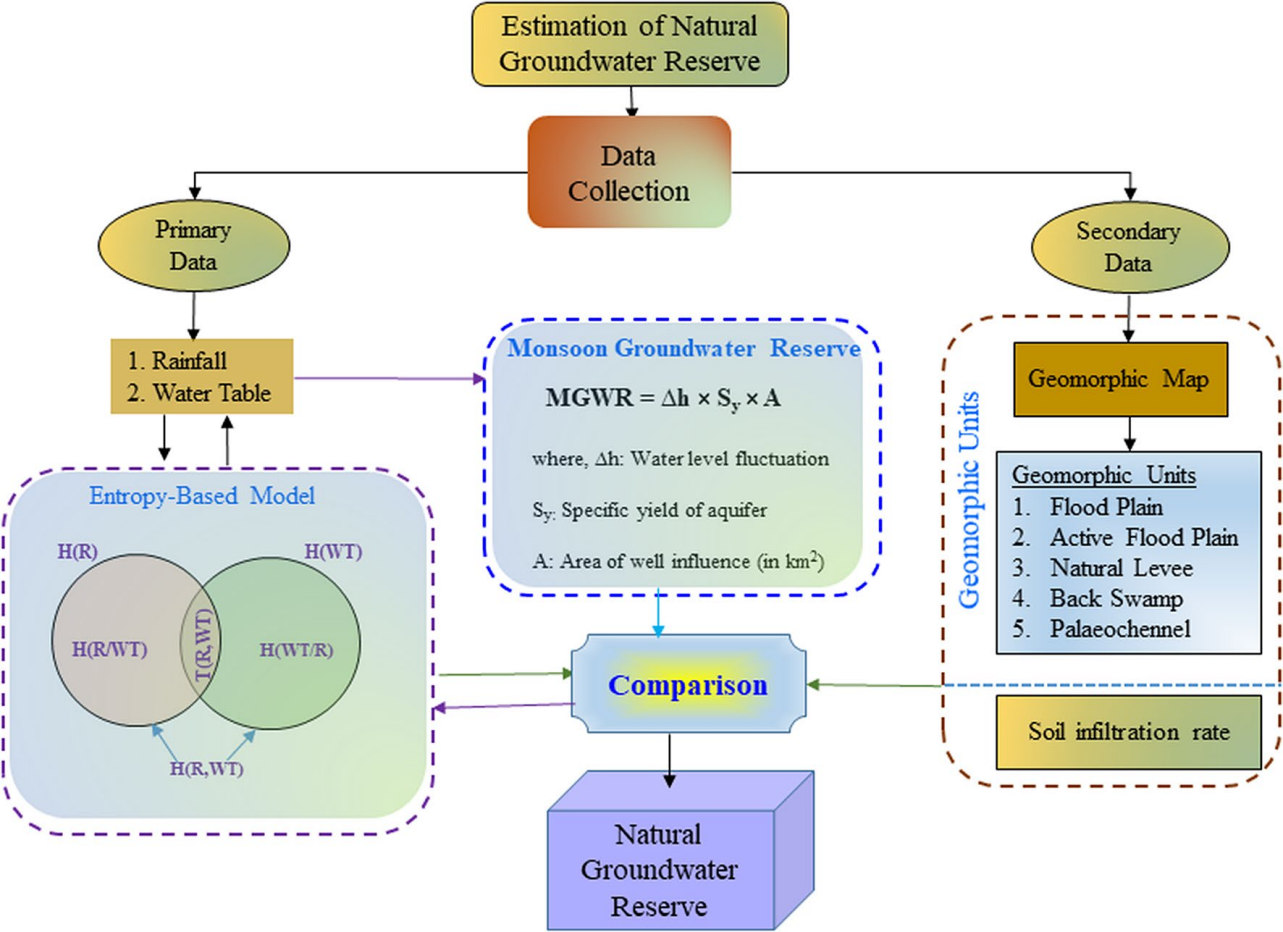
Flowchart for estimating groundwater reserve using an information-based model at the proximity of a morphodynamic system in a part of the Ganga basin, Northern India.

Depth to groundwater table (WT) fluctuates due to the rainfall (R) recharge. Both the WT and R are random variables. Then the marginal entropies [i.e., H(R) and H(WT)] are estimated in the probability distribution, and categorized as the potential information of these variables ^27,28^. Joint entropy, H (R, WT) is calculated as the total information having in the measurements of WT and R, as shown in Fig. 2. Then the transinformation [T (R, WT)] gain measures the reduction of entropy by splitting the dataset according to a probability space of random variable, which provides the physical process of the hydrogeological system. A larger information gained suggests a lower entropy of the analysed data and/ gaining the information of the physical processes. It is calculated as the reduction in the original uncertainty of the measurement of WT due to the knowledge gained in the measurement of rainfall (R). The discrete forms of these entropies are described as^27,28^.

4 $$H(R)\, = \,\, - \,\sum\limits_{i\, = \,1}^{n} {\,p(R_{i} )\,\ln \,p(R_{i} )}$$

5 $$H(WT)\, = \,\, - \,\sum\limits_{j = \,1}^{m} {\,p(WT_{j} )\,\ln \,p(WT_{j} )}$$

6 $$H(R,WT)\, = \,\, - \,\sum\limits_{i\, = \,1}^{n} {\sum\limits_{j = 1}^{m} {p(R_{i} ,WT_{j} )} \,\ln \,p(R_{i} ,WT_{j} )}$$

7 $$T(R,WT) = H(R) + H(WT) - H(R,WT)$$

where, R and p(R_i_): discrete variable of rainfall and probability of R, respectively.

WT and p(WT_j_): discrete variable of depth to water level and probability of WT, respectively.

p (R_i_, WT_j_): joint probability of R_i_ and WT_j_. R_i_, i = 1, 2, …, n; and WT_j_, j = 1, 2, …, m, defined in the same probability space.

Then, the percentage of rainfall recharge, R_e_ (%) is the ratio of T (R, WT) and H(R) multiplying 100, which is represented the natural groundwater reserve due to the rainfall as described in the following equation.

8 $$R_{e} (\% ) = \frac{T(R,WT)}{{H(R)}} \times 100$$

A 2-D contingency table (Table S1)^28,35–37^ is essential to estimate the values of H(R), H(WT), and T (R, WT), as presented in Table S1. To construct this table, rainfall data comprised the u-class intervals, whereas the depth to water table data was assumed to be the v-class intervals. Then, the joint frequency of the rainfall and depth to water level data categorised by (i, j) is denoted by f_ij_, i = 1, 2, . . . , u; and j = 1, 2, . . . , v, where ‘i’ considered the column, and ‘j’ considered the row. In deciding the number of classes, the 2^ k^ rule [2^ k^ ≥ N] was utilized in the characterizing data sets^38^. This rule was used to reduce the error of recharge percentage by changing the number of classes (k) and class intervals of the data (N) at the well site of the study area.

### Monsoon groundwater reserve

Monsoon groundwater reserve is estimated using the water table fluctuation (WTF) method. It is based on the principle that rises in water level in shallow (unconfined) aquifer is due to groundwater recharge arriving at the water table^8,39^. Monsoon groundwater reserve (MGWR) was obtained from the following equation.

9 $${\text{MGWR }} = \, \Delta {\text{h }} \times {\text{ S}}_{{\text{y}}} \times {\text{ A}}$$

where, ∆h: Water level fluctuation between pre and post-monsoons, S_y :_ Specific yield of the aquifer [fraction], and A: Geographic area of calculation of groundwater reserve (in km^2^) obtained by Theissen polygon method^40^.

Then the MGWR was estimated using the above equation at 50 monitoring well sites (mainly at each Thiessen polygon) during the years 2012 and 2013. Because the monthly groundwater level data in a dense monitoring well network were available, as shown in Fig. 1c. The idea of the Thiessen polygon method was that the zones addressing every groundwater measure point were characterized by drawing lines between neighbouring points on a map. The perpendicular bisectors were developed to lines joining each measurement point with individuals proximately surrounding it. These bisectors formed a succession of polygons, each polygon containing one measuring point^40^. Groundwater reserves had been estimated at all observation well sites, and the area of influence of each well site was calculated using the polygon method in ArcGIS (10.4). In total, 50 polygons were created. The minimum area of ~ 3.19 km^2^ was influenced by the well (W-40) located in the flood plain, and the maximum area of 20.12 km^2^ by the well (W-47), as shown in Figs. 1c, 4. The estimated natural groundwater resources at each polygon had been also utilized to compare with the results obtained from the information-based model.

### The data

#### Primary data

Monthly groundwater level at 50 observation wells of a dense monitoring network from February 2012 to March 2014 were accessed and utilized for the analysis. The monthly rainfall data were also collected from January 2010 to December 2019 (http://hydro.imd.gov.in/hydrometweb/). It had been observed that the rainy season, in general, continues from mid-June to the end of September, which receives the Southwest monsoon and accounts for about 86.2% of the total average rainfall (~ 872.8 mm). It gets an average normal monsoon rainfall of about 752.3 mm/year from the year 2010–19 (Fig. S1a). The seasonal rainfall of the year 2010–2019 had been calculated as around 752 mm, as presented in Table S2, and used for our analysis.

The transformation value, T (R, WT) of the rainfall and WT, along with the marginal entropy, H(WT) of the groundwater level at the individual well, had been estimated with the aid of Eqs. (4,5,6,7). Then the percentage of rainfall as a natural groundwater recharge was calculated with the help of Eq. (8). In addition, the specific yield values of 8–10% were considered for the sandy loam and sandy clay loam, whereas 7–8% for the clay loam of the study area (Fig. S2) as per the GEC norms^41^. These values were considered to estimate the MGWRs at these well areas using the Theissen polygon method^40^.

#### Secondary data

In addition, the geomorphic map of the study area (Fig. 1c) was utilized to compare the estimated recharge at the well site. Figure 1c shows five different units such as active flood plain (~ 6 km^2^), palaeochannel (~ 55 km^2^), back swamp (~ 65 km^2^), natural levees (~ 91 km^2^), and flood plain (~ 304 km^2^). This map was used to relate the estimated groundwater reserves in the various geomorphic units, as shown in the flowchart (Fig. 2).

## Results and discussion

### Rainfall

In the study area, about 6.8% of the annual rainfall precipitated in winter (October to February), about 7.0% in summer (March–May), and about 86.2% in the Southwest monsoon period (June–September), as presented in Table S2. It had shown that the rainfall of around 752 mm occurred during the southwest monsoon. This monsoon rainfall was comparatively more (> 220 mm)^17^, and was also found only one stretch for the monsoon period (Fig. S1b). Rangarajan and Athavale^17^ studied based on tracer technique to estimate the natural groundwater recharge in different hydrogeological provinces in India. Their results suggest that the linear relation between rainfall and natural recharge exists in the major hydrogeological units of India. The minimum precipitation is required to initiate the rainfall recharge due to the soil characteristics and hydrogeological conditions of the aquifer. It has been illustrated that the minimum values of the rainfall needed for the recharge, are 40 mm for alluvial areas, 220 mm for sediments, 255 mm for granite, and 355 mm for basaltic areas^17^. Thus, the natural groundwater recharge had been calculated during the Southwest monsoon when around 86.2% (~ 752 mm) of the annual rainfall occurred in the study area (in Table S2).

### Groundwater level and its fluctuation

Monthly groundwater levels were monitored at 50 open wells (depth range: 4.25 to 14.00 m, below ground level (bgl)), during the first week of every month from February 2012 to March 2014. The well location is shown in Fig. 1c. The reduced levels varied from 45.90 to 62.40 m, amsl, with a mean of 51.92 m, amsl (Table S3). Groundwater level varied from 39.01 to 59.10 m, amsl, with an average of 47.04 m, amsl during the pre-monsoon (May 2012), whereas it was varied from 44.57 to 61.63 m, amsl, with an average of 50.35 m, amsl during the post-monsoon (October 2012). The water level raised an average of 3.31 m. The maximum rise was observed at Parsa Bazar (well: 26), and the minimum rise at Dalluchak (well:29), shown in Fig. S3. There was no decline in groundwater level after the monsoon, but the groundwater level raised more than 4.00 after the monsoon in the central northern and southern parts.

**Figure 3:**
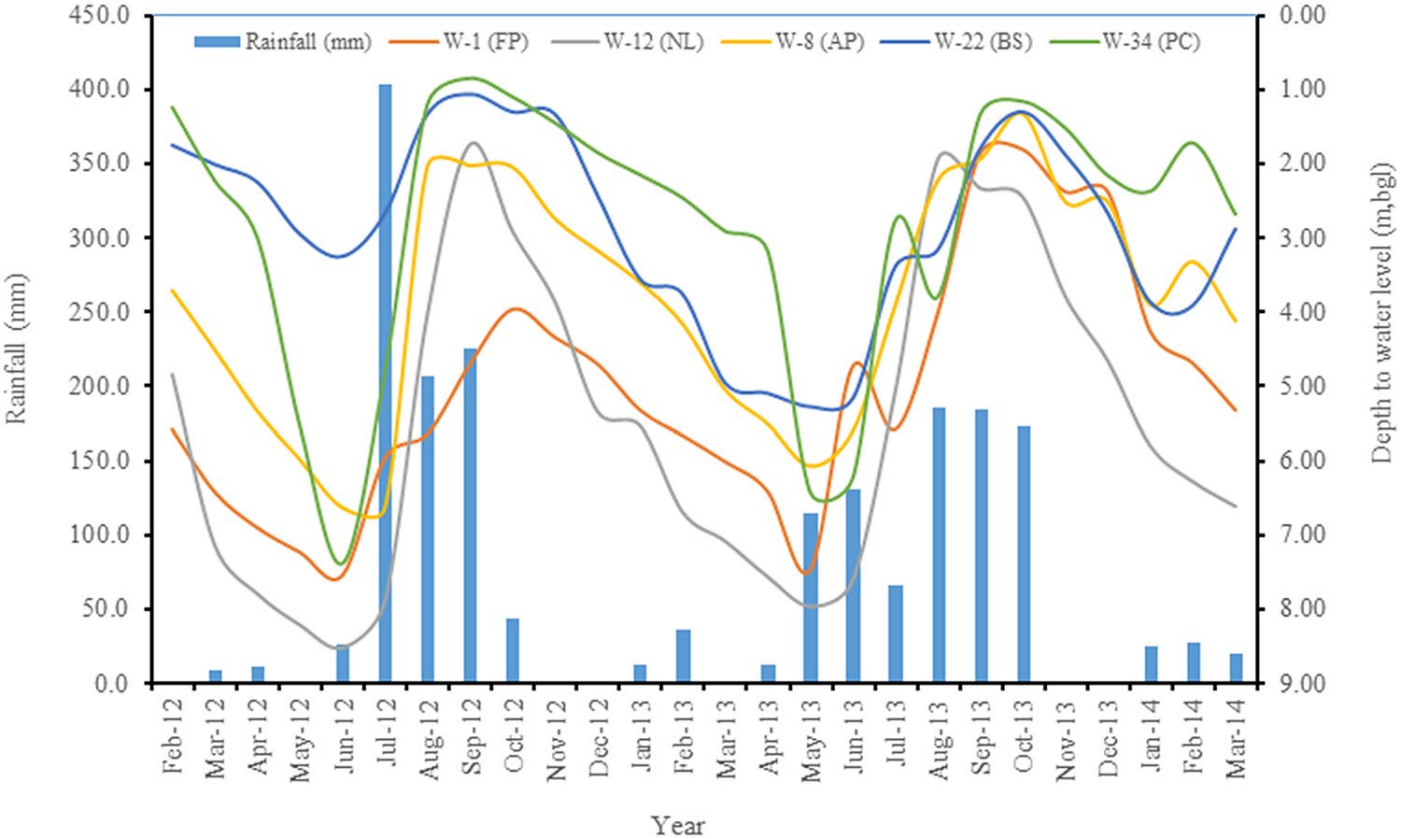
Well hydrographs corresponding rainfall in a morphodynamic system in a part of the Ganga basin of Northern India [FP: Flood Plain, NL: Natural Levees, AP: Active Flood Plain, BS: Back Swamp, and PC: Palaeochannels].

In this river basin, where groundwater occurs in unconfined (shallow) aquifers, the rise in the groundwater table is a direct consequence of rainfall, particularly in the rainy season^42^. This rise in groundwater level at the well site is a characteristic feature of the unsaturated zone^43,44^. So, there exists a definite relationship between the rainfall and the depth to water table for a particular region. The well hydrographs corresponding rainfall were made, as shown in Fig. 3. This figure had shown that the groundwater level at the wells located in an active flood plain (W-8), natural levees (W-12), back swamp (W-22), and palaeochannel (W-34) are strongly correlated with the rainfall after 2-month lags having the correlation coefficients of −0.65, −0.76, −0.63, and −0.50, respectively. In comparison, the well (W-1) located at flood plain in the extreme north-western part of the study area responded after a 3-month lag. It was due to the confluence of rivers Son and Ganga. Therefore, cross-correlation among rainfall and groundwater level at the individual well was carried out. It had been observed that the well (W-9) and W-8 were responded after 1- and 2- month lags of rainfall with the cross-correlation coefficients of −0.62 and −0.65, respectively, in an active flood plain located in the north-western part. In the back swamp, the wells were responded to after 1–2 month lags in the range of correlation coefficients of −0.49 to −0.77, with an average of −0.64. The wells were responded to within 1–3 month lags in the flood plain, and palaeochannel areas, with the average cross-correlation coefficients of −0.66 and −0.58, respectively. But the wells located in natural levees responded within 1-month lags after the rainfall except for the wells at W-4 (Korhar), W-12 (Khaspur), W-21 (Patna Law College), and W-23 (Sornapur). It indicates that the well hydrographs were directly responded after the rainfall, where the natural recharge had been taken place.

### Information-based recharge estimation

The shallow groundwater occurs in unconfined aquifers, particularly in a morphodynamic system in a part of the Ganga basin of Northern India. The water level rise in the rainy season was an immediate result of precipitation, when the groundwater draft was comparatively less. The water table rise at a specific place was a typical feature of the saturated zone^39^. Thus, the information-based model had been hypothesized and utilized to assess natural groundwater recharge in this morphodynamic system. For this, the marginal and joint probabilities were estimated separately using a 2-D contingency table^27,28^ for both the monthly and SW monsoon data. In total, 8 events of the SW monsoon data, and 26 events of the monthly data were used to construct the contingency tables. An illustration of a 2-D contingency table for the monthly data of well W42 (village: Tishkhora) had been prepared, as presented in Table 1. The rainfall data had been categorized into 4 class intervals in the ranges of 0–100, 100–200, 200–300, and 300–410 mm, whereas the groundwater level data into 5 class intervals in the ranges of 0–2, 2–4, 4–6, 6–8, and 8–10 m, bgl based on the 2^ k^ rule^38^ to reduce the error of Re (%) estimation at the well site. The joint frequency (i, j) denoted by f_i,j_ (where i = 1,2,…,4; and j = 1,2,.., 5), were considered as the column (rainfall, i) and the row (water level, j). The f_i_. and f_j_. were referred to as the marginal frequencies of these two variables, respectively. The marginal entropies of rainfall (1.290 bits) and water level (0.961 bits), joint entropy (2.144 bits), and transinformation (0.107 bits) were estimated, as presented in Table 1. During the SW monsoon, the natural recharge at the well (W-42) site was calculated with the help of Eq. (8), and found about 8.30% of the rainfall. Similarly, it was estimated at the remaining well sites, as presented in Table S4. It indicates that the wells located at Phulwari (W-28), Dalluchak (W-29), and Raghopur (W-40) were not inconsiderable response due to the precipitation. These wells were independent of natural groundwater recharge, but the water level fluctuation might be responded to due to the lateral flows, and human intervention. Except at these well sites, the transinformation was measured an average of 0.238 bits in the monthly data, whereas average information of 0.662 bits in the SW monsoon season. It shows that the precipitation in the SW monsoon season is more dominant in influencing the natural groundwater recharge in the study area. It had been observed that during the monsoon season, the average information gained was around 1.031, 0.874, 0.610, 0.619, and 0.643 bits in the active flood plain, back swamp, palaeochannel, flood plain, and natural levees, respectively. Annual average rainfall recharge was estimated at around 18.49% (Table S4), whereas it was an average of 34.73% of the SW monsoon season. It had been found that the natural recharge was comparatively more in the active flood plain (~ 38.41%) than the natural levees (17.03%) in the monsoon season.

**Table 1:** Absolute frequency 2-D contingency table for rainfall (R in mm) and depth to water table (WT, m, bgl) at well W 42 (village: Tishkhora) located in the flood plain of a morphodynamic system in a part of the Ganga basin of Northern India.

	WT (m, bgl)	Rainfall (R) in mm	i = 1	i = 2	i = 3	i = 4	Total	Pj	Log Pj	Pj log Pj	H (WT)
		Rainfall(mm)	0–100	100–200	200–300	300–410					
Marginal entropy	j = 1	0–2	11	3	2	0	16	0.615	−0.700	−0.431	0.961 bits Obtained From the Eq. (5)
	j = 2	2–4	7	2	0	1	10	0.385	−1.379	−0.530	
	j = 3	4–6	0	0	0	0	0	0.000			
	j = 4	6–8	0	0	0	0	0	0.000			
	j = 5	8–10	0	0	0	0	0	0.000			
		Total	18	5	2	1	26				
		P_i_	0.692	0.192	0.077	0.038					
		Log Pi	−0.531	−2.379	−3.700	−4.700					
		Pi*Log Pi	−0.367	−0.457	−0.285	−0.181					
		H (R)	1.290 bits obtained from the Eq. (4)								
		Pi,j	0.423	0.115	0.077	0.000					
			0.269	0.077	0.000	0.038					
			0.000	0.000	0.000	0.000					
			0.000	0.000	0.000	0.000					
			0.000	0.000	0.000	0.000					
		Pi,j *Log Pi,j	−0.525	−0.359	−0.285	0.000					
			−0.510	−0.285	0.000	−0.181					
			0.000	0.000	0.000	0.000					
			0.000	0.000	0.000	0.000					
			0.000	0.000	0.000	0.000					
Joint entropy	H (R, WT) =	2.144 bits obtained from the Eq. (6)									
Transformation	T (R, WT) =	0.107 bits obtained from the Eq. (7)									
Natural recharge (%)	R (%) =	8.30 obtained from the Eq. (8)									

The spot measurements of natural recharge using the tracer techniques at a few alluvium sites located in Tamil Nadu, Andhra Pradesh, West Bengal, Bihar, Uttar Pradesh, Gujrat, Rajasthan, Haryana, and Punjab had been carried out by several researchers^17,45–47^. Their results indicate that the range of natural recharge varied from 10.90 to 19.70% of seasonal rainfall, with an average of 13.86%. In the study area, the recharge zones had been identified considering the soil texture^48,49^. It had been found that the monthly recharge of rainfall estimated ~ 20% of the infiltration factor was used for the groundwater flow model, which had shown reasonably matched with the calibrated well hydrographs^50^. The estimated average natural recharge in the study area using information-based theory was an average of 18.49% of the rainfall (Table S4), which is quite acceptable. Because it was also estimated in the range of 10.90 to 19.70% of seasonal rainfall at the same typical hydrogeological conditions in the states of Uttar Pradesh, Bihar, and West Bengal^17,46,47^. It was noted that the estimated joint entropies at the well sites were not systematic due to the non-uniformity of rainfall and various morphological units in the study area. Therefore, the measurement of natural recharge using this entropy-based model will be more accurate, if the precipitation is recorded at each nearby well site.

### Estimation of monsoon groundwater reserve

**Figure 4:**
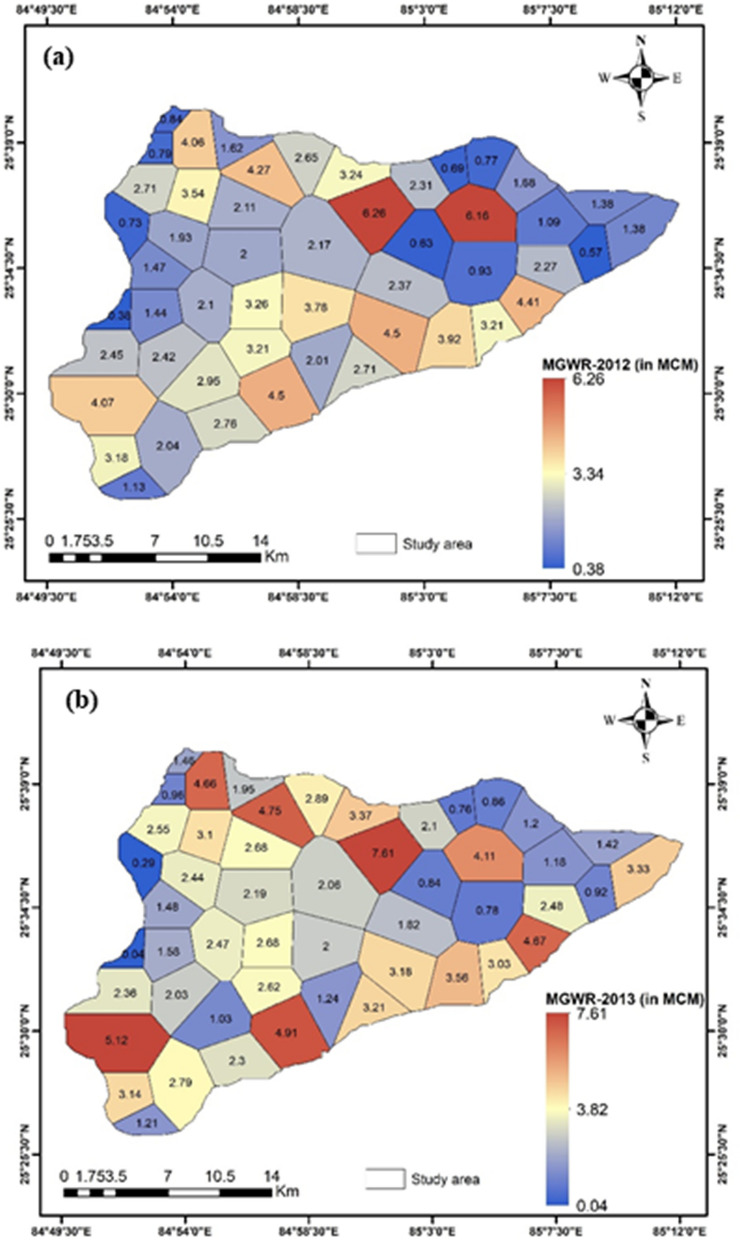
Thiessen polygons and the estimated monsoon groundwater reserve (in MCM) at each polygon in a part of the Ganga basin of Northern India for the year of (**a**) 2012, and (**b)** 2013 (V.A.: drawn this figure using the Thiessen polygon tool in ArcGIS ver.10.4, http://www.esri.com).

The estimated MGWR results present that the lowest value was about 0.04 MCM in 2013 at the influence well area (at W-40 in flood plain) of Raghopur village located in the western part (Fig. 4b, Table S5), and the highest value was around 7.61 MCM in 2013 in the area of Murarchak village (at W-16, flood plain) located in the northern part of the study area. In comparison, the maximum and minimum of 6.26, and 0.38 MCM were encountered at these well sites in 2012. Then, the MGWR of the entire study area had been estimated in the range of 121.38 -123.03 MCM for two hydrogeological seasons. The maximum MGWR of 123.03MCM was evaluated in 2012, when the contribution of monsoon rainfall was about 97.0% of the annual rainfall (~ 933.0 mm). It was around 121.38 MCM for the year 2013 due to the monsoon precipitation of 854.9 mm in the study area.

### Cross plot of soil infiltration rate and natural recharge

**Figure 5:**
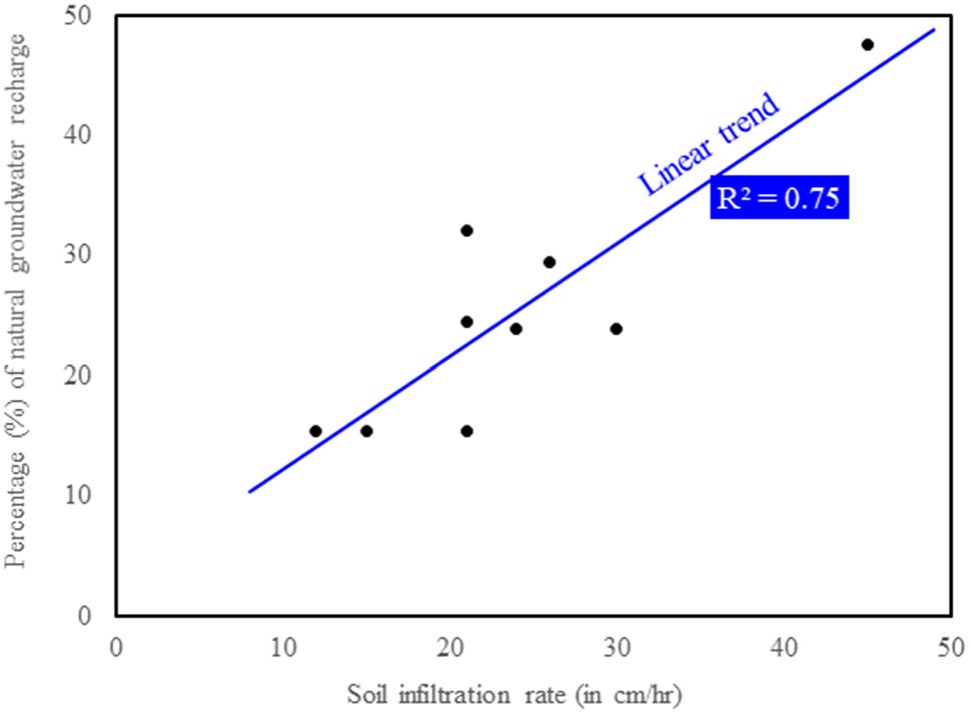
Cross plot the percentage of natural groundwater recharge with soil infiltration rate in a part of the Ganga basin during the monsoon period.

The soil infiltration experiment was carried out at nine sites in barren lands of the study area (Fig. 1c). The infiltration rates ranged from 12.0 to 45.0 cm/hr, with an average of 23.9 cm/hr. A cross plot of the estimated natural groundwater recharge with the infiltration rate had been made. A good positive correlation between the infiltration rate and the percentage of natural groundwater recharge was obtained with R^2^ = 0.75 in the monsoon period, as shown in Fig. 5.

### Recharge variability in morphodynamic units

**Figure 6:**
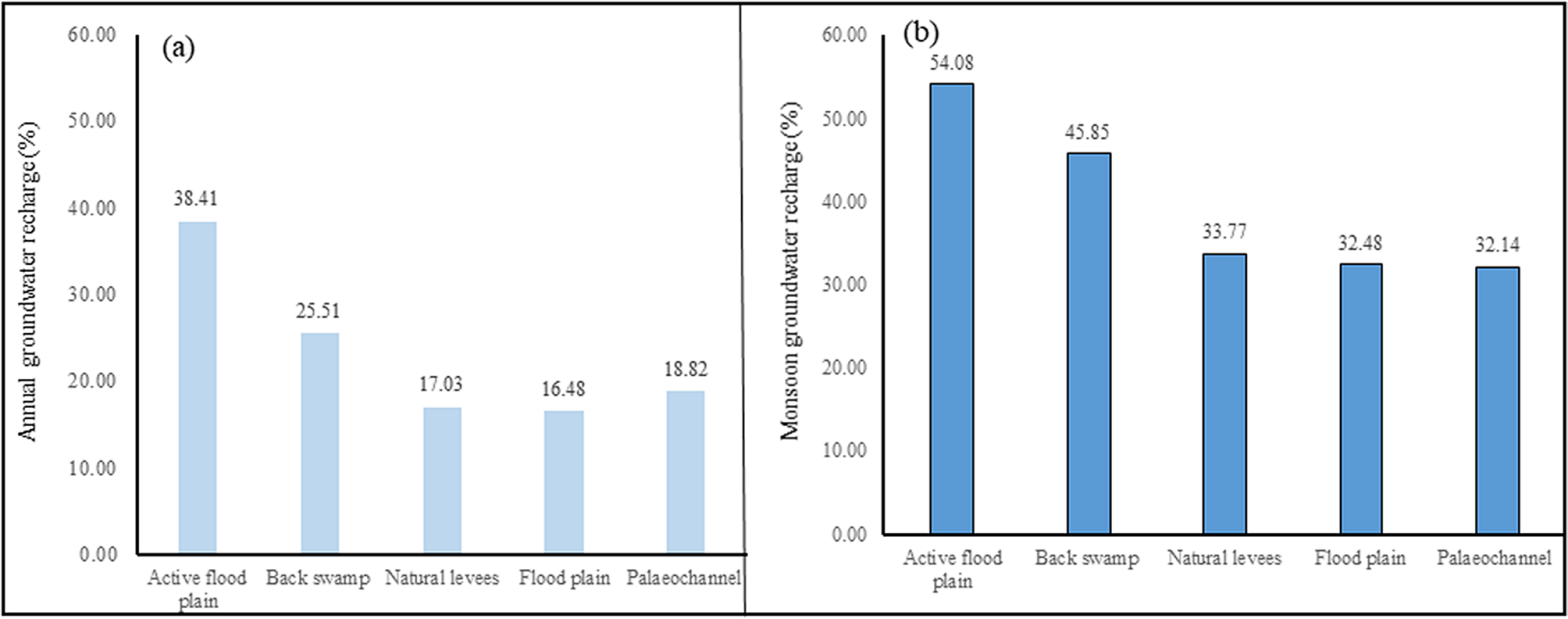
Percentage (%) of natural groundwater recharge due to the (**a**) annual, and (**b**) monsoon rainfall in the geomorphic units in a part of the Ganga basin.

Geomorphic features combined with structures and lithology controls allow the occurrence, movement, and quality of groundwater. It plays an important role in the identification of favourable zone for groundwater recharge. In the study area, the morphological units such as active flood plain (~ 6 km^2^), palaeochannel (~ 55 km^2^), back swamp (~ 65 km^2^), natural levees (~ 91 km^2^), and flood plain (~ 304 km^2^) exit. The active flood plain associated with high porosity and permeability having a variable thickness of the soil is capable of groundwater recharge. It had been observed that the wells located at natural levees (W-28), back swamp (W-29), and flood plain (W-40) were not responding significantly due to the rainfall. It had found that the average percentage of estimated natural groundwater recharge at active flood plain was ~ 38.41(%) of annual rainfall, and ~ 54.08% of monsoon rainfall. In contrast, it was ~ 18.82% and ~ 32.14%, respectively, in palaeochannel, which is the main charter in terms of geomorphology in this river basin. In the flood plain (area: ~ 304 km^2^), mainly located in the central, southern, eastern, and western parts of the area, the percentages of natural recharge were ~ 16.48% of annual rainfall and ~ 32.48% of monsoon precipitation (Fig. 6). It had been observed that the active flood plain was more dominant for the natural groundwater recharge comparatively the natural levees.

### Natural groundwater recharge

The percentage of natural groundwater recharge was estimated using the developed entropy-based model for the data of the year 2012–2014, because monthly groundwater level data were available at 50 well sites within a dense monitoring well network in the research area. It is the general practice when the percentage of recharge is known, used for the estimation of natural groundwater recharge simply measuring the precipitation of that area. This approach was also applied in the tritium injection method by several researchers^17,18^. The calculated natural groundwater recharge using the information-based model varied from 95.25 to 184.11 MCM/monsoon rainfall, with an average of 127.98 MCM for the period of 2010–2019 (Fig. S4), which was almost the same order reported by CGWB and other researchers in the study area^49,50^. Also, it was in the equivalent of about 121.46 MCM/monsoon rainfall estimated by the water level fluctuation method. The minimum reserve of 95.25 MCM was estimated in 2015, when the monsoon and annual rainfall were 560.0 mm, and 608.8 mm, respectively. The maximum of 184.11 MCM in the year 2011, when the monsoon and annual rainfall were 1082.4 mm, and 1161.9 mm, respectively. But the natural groundwater reserve was estimated at about 137.91 MCM during the monsoon period when the rainfall was 810.8 mm in 2019.

**Figure 7:**
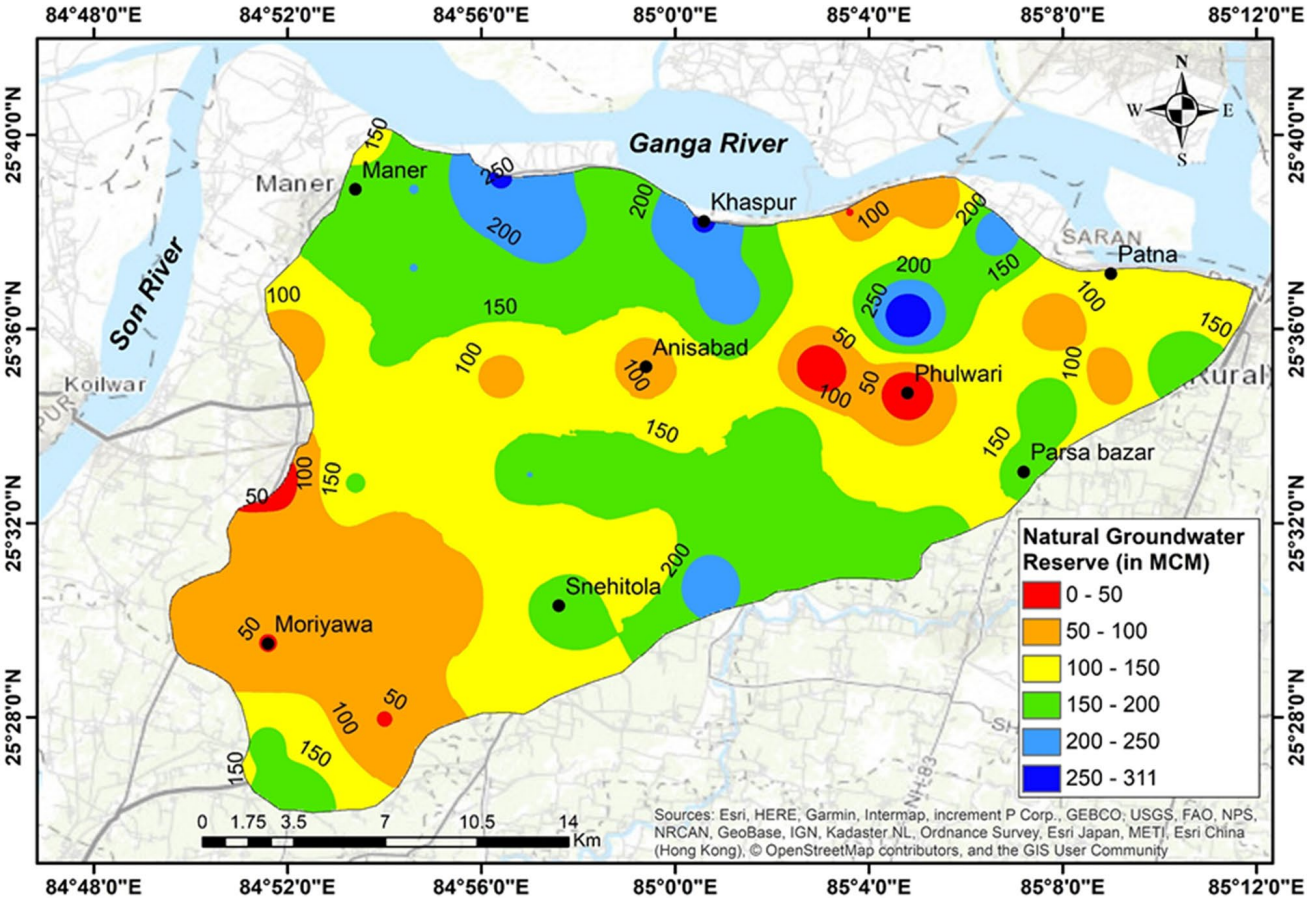
Groundwater reserve in a morphodynamic system at a part of Ganga basin, Northern India during the monsoon precipitation of the year 2019 (N.C.M.: sketched this figure with the help of ArcGIS Desktop 10.4, http://www.esri.com).

The spatial variability of natural groundwater recharge during the SW monsoon season in 2019 had been estimated by knowing the percentage of recharge and precipitation of that area, as shown in Fig. 7. It had been observed that the recharge varies spatially depending upon the influence factors such as geomorphology, soil, and drains pattern^23^. Its’ magnitude was nicely collaborated, with the infiltration rates of clay, sand, and sandy clay^48^. It had been noticed that the natural reserve was negligible at the well areas located at Phulwari (W-26) and Dalluchak (W-29), which lay in natural levees and back swamp, respectively, at the middle-east of the study area. The reserve was also negligible at Raghopur (W-40), located in the extreme western part. Broadly, the natural groundwater reserve was in the range of 150–311 MCM in the northern and southern parts (Fig. 7), whereas it was in the range of 100–250 MCM in the monsoon period of 2019 at the middle part. But it was about 50–100 MCM in the part of Patna city, and the south-western part. This estimated groundwater reserve could be utilized to develop groundwater flow modelling and budgeting of water resources in this area.

## Conclusion

The information-based model has been hypothesized and developed to estimate natural groundwater recharge using simply measured groundwater levels in shallow aquifers and rainfall. It has been applied to evaluate groundwater reserve in a morphodynamic system in a part of the Ganga basin, Northern India. The results show that the average natural recharge rate is about 246 mm/ monsoon rainfall, and 32.65% of the seasonal monsoon rainfall. The estimated recharge is well-correlated with the soil infiltration rate, as well as the monsoon groundwater reserve (MGWR) obtained from the water level fluctuation method. It has been found that the natural groundwater recharge rates in an active flood plain, back swamp, natural levees, flood plain, and palaeochannel of the study area are about 54.08, 45.85, 33.77, 32.48, and 32.14% of the seasonal rainfall, respectively. The estimated natural groundwater reserve varies from 95.25 to 184.11 MCM, with an average of 127.98 MCM during the monsoon period from 2010 to 2019. The reserve is nicely manifested with the soil infiltration rate and the geomorphic units. Although other factors such as vegetation, topography, geology, and climate control the recharge, and therefore, there is an impact of the selecting technique for estimating groundwater recharge. But the estimated natural groundwater recharge using this developed information-based model will help to improve groundwater management practices such as the construction of artificial recharge structures, and simulating groundwater flow model for the budgeting of water resources on a regional scale at a glance. This approach could be adopted for estimating natural groundwater reserves at any hydrogeological setting under diverse meteorological conditions without any hydrogeological property.

## Supplementary Information

Supplementary Information.

## Data Availability

The data source: http://cgwb.gov.in/AQM/Pilot/Patna%20District,%20Bihar-Final.pdf and the analyzed information during the current study are available from the corresponding author on reasonable request.
