# Author Correction: Assessment of natural groundwater reserve of a morphodynamic system using an information-based model in a part of Ganga basin, Northern India

**DOI:** 10.1038/s41598-022-26436-z

**Published:** 2022-12-29

**Authors:** N. C. Mondal, V. Ajaykumar

**Affiliations:** 1 Earth Process Modeling Group, CSIR-National Geophysical Research Institute, Hyderabad, 500 007 India; 2 Academy of Scientific and Innovative Research (AcSIR), Ghaziabad, 201 002 India

Correction to: *Scientific Reports* 10.1038/s41598-022-10254-4, published online 13 April 2022

The original version of this Article contained an error in the legend of Figure 1.

“Study area along with the well and infiltration sites on the geomorphic map in a part of the Ganga basin, Northern India (N.C.M.: sketched this figure with the help of ArcGIS Desktop 10.4, http://www.esri.com).”

now reads:

“Study area along with the well and infiltration sites on the geomorphic map in a part of the Ganga basin, Northern India (N.C.M.: sketched this figure with the help of ArcGIS Desktop 10.4, http://www.esri.com, CGWB, 201548).”

In addition, in the Materials and methods section, under the subheading ‘The data - Primary data’,

“Groundwater level was measured monthly at 50 observation wells of a dense monitoring network from February 2012 to March 2014, and the monthly rainfall data were also collected at the Patna rain gauge station from January 2010 to December 2019.”

now reads:

“Monthly groundwater level at 50 observation wells of a dense monitoring network from February 2012 to March 2014 were accessed and utilized for the analysis. The monthly rainfall data were also collected from January 2010 to December 2019 (http://hydro.imd.gov.in/hydrometweb/).”

Also, this Article contained an error in Reference 48, which was incorrectly given as:

Central Ground Water Board (CGWB). Report on pilot project on aquifer mapping in Maner-Khagaul area, Patna District Bihar (Watershed -GNDK013), p. 260 (2015).

The correct reference is listed below:

Central Ground Water Board (CGWB). Report on pilot project on aquifer mapping in Maner-Khagaul area, Patna District Bihar (Watershed -GNDK013), p. 260 (2015). http://cgwb.gov.in/AQM/Pilot/Patna%20District,%20Bihar-Final.pdf

Furthermore, the Acknowledgements section was incomplete.

“Prof. V.M. Tiwari, Director of CSIR-National Geophysical Research Institute, Hyderabad, has encouraged and permitted to publish this article (Ref. No: NGRI/Lib/2021/Pub-18). The DST-NGP (GoI), New Delhi (Ref. No.: NRDMS/GRACE/8342/NC Mondal/2020(G), January 21, 2021) has funded this research work. Authors are thankful to them.”

now reads:

“Prof. V.M. Tiwari, Director of CSIR-National Geophysical Research Institute, Hyderabad, has encouraged and permitted to publish this article (Ref. No: NGRI/Lib/2021/Pub-18). The DST-NGP (GoI), New Delhi (Ref. No.: NRDMS/GRACE/8342/NC Mondal/2020(G), January 21, 2021) has funded this research work. Central Ground Water Board (CGWB), Ministry of Jal Shakti, Department of Water Resources, River Development and Ganga Rejuvenation (GoI) has generated the hydrogeological data. Authors are thankful to them."

Lastly, the Data availability section was incomplete.

“The data sets both generated and analysed during the current study are available from the corresponding author on reasonable request.”

now reads:

“The data source: http://cgwb.gov.in/AQM/Pilot/Patna%20District,%20Bihar-Final.pdf and the analyzed information during the current study are available from the corresponding author on reasonable request”

The original Article has been corrected.

