# Supplementary material for: Assessment of natural groundwater reserve of a morphodynamic system using an information-based model in a part of Ganga basin, Northern India
Source: Sci Rep. 2022 Apr 13;12:6191. doi: 10.1038/s41598-022-10254-4 (PMC9008018; doi:10.1038/s41598-022-10254-4)
Supplement: Supplementary file 1 — Supplementary Information. [file 41598_2022_10254_MOESM1_ESM.pdf]

Assessment of natural groundwater reserve of a morphodynamic system using an information-based model in a part of Ganga basin, Northern India

N.C. Mondal[a,b*] and V. Ajaykumar[a,b]
[a]Earth Process Modeling Group, CSIR-National Geophysical Research Institute, Hyderabad 500 007, India
[b]Academy of Scientific & Innovative Research (AcSIR), Ghaziabad - 201 002, India
Ph.: + (91)-40-27012608, Fax: + (91)-40-23434651
***Corresponding author**: mondal@ngri.res.in; ncmngri@gmail.com

Supplementary material

S1: About the study area

S2: Table S1 Two dimensional contingency chart (frequency)

S3: Table S2. Season wise normal rainfall in a morphodynamic system in a part of the Ganga basin of Northern India

S4: Table S3. Detailed well inventory in a morphodynamic groundwater system in a part of the Ganga basin, Northern India

S5: Figure S3. Water level contours map of May 2012, and its fluctuation

S6: Table S4. Estimated percentage natural recharge (%) using information-based model for the monsoon data at the shallow well areas at a morphodynamic system in a part of the Ganga basin

S7: Table S5. Estimated the monsoon groundwater reserves

S8: Figure S4. Natural groundwater reserve for the period of 2010-2019

S1: About the study area

Study area. The study area covers an area of 521 km^2 spreading in the parts of 7-blocks of Patna district, Bihar in Middle Ganga Plain (MGP) in the Northern India. The area lies between longitudes: $84^{0}49^{/}12^{//}$ E to $85^{0}13^{/}12^{//}$ E and latitudes:$25^{0}25^{/}12^{//}$ N to $25^{0}40^{/}$’48 $^{//}$N (**Fig. 1**). It falls in the Survey of India Topo-sheets 72G/2 and 72C/14,15. Topography varies from about 45.45 m to 69.00 m, amsl with a general slope from S-W to N-E and N-directions with minor variations. The mighty River Ganga forms the northern boundary, and other rivers (i.e., Son and Punpun) are draining just outside the area. It falls under a subtropical climate. Winter and summer seasons start from the month of November and continues up to February, and from March and continues up to April, respectively. But the rainy season started from June and ended in the month of October. Maximum rainfall occurs due to the S-W monsoon and accounts for about 86.2% of total rainfall during 2010-2019. An average normal monsoon rainfall is of about 752.3mm/year, as shown in **Fig.S1a**. Soil type is mainly clay loam (**Fig.S2**). But sandy loams with clay loam at some places are also predominant soils in this area. Geomorphic map of the study area (**Fig.1c**) shows five different units (i.e., flood plain, active flood plain, natural levee, back swamp, and palaeochannels) in the study area.

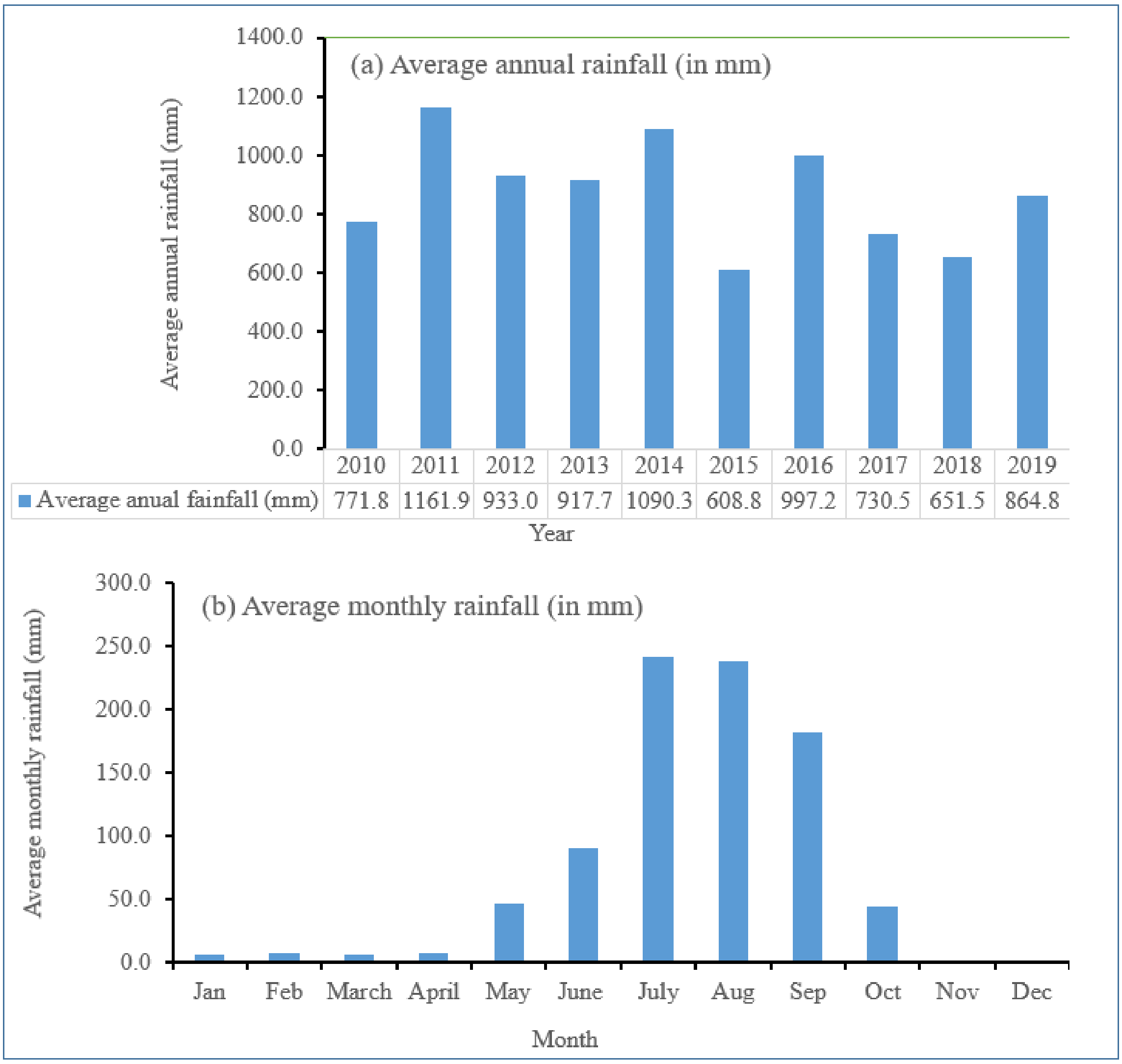

Figure S1. Showing (a) average annual rainfall (in mm), and (b) average monthly rainfall (in mm) in a morphodynamic system in a part of the Ganga basin of Northern India

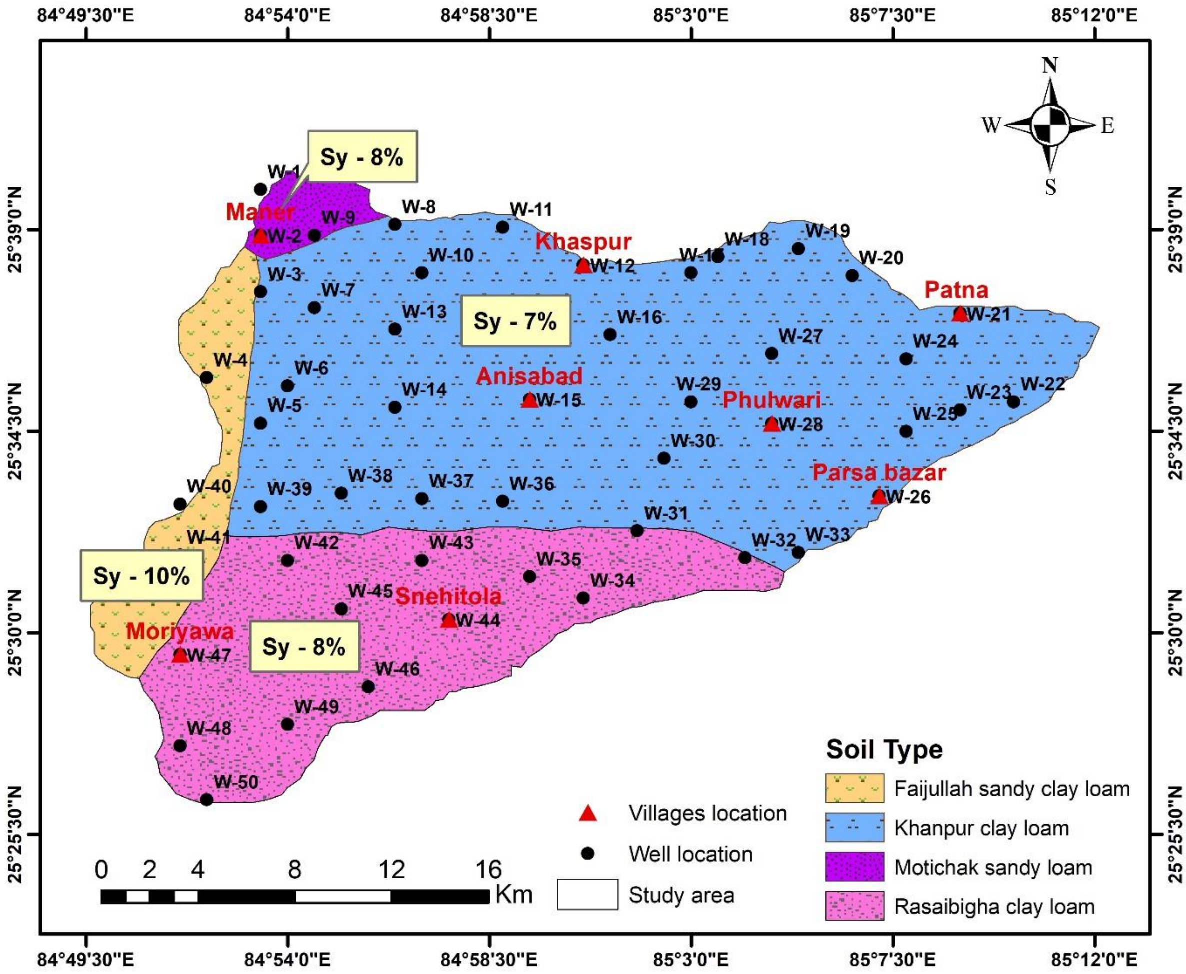

Figure S2. Soil types of a morphodynamic system in a part of the Ganga basin of Northern India (V.A.: drawn this figure using ArcGIS ver.10.4, http://www.esri.com,http://cgwb.gov.in/AQM/Pilot/Patna%20District,%20Bihar-Final.pdf)

In the area immediately south of River Ganga, the aquifers commence from an average depth of 40 m and continue downward up to 300 m as per the explored borehole information (Mondal et al., 2014). However, the thickness of pile sediments is more than 700 m forming a multi-aquifer system. The aquifers are separated by clay beds of varying thicknesses (Mondal et al., 2015). These aquifers are under exploitation for irrigation and domestic needs.

Groundwater mainly occurs under unconfined conditions at the shallower depths, but under the semi-confined to the confined conditions in a deeper level. The depth to the groundwater at the unconfined aquifers ranges between 1.32 and 9.69 m bgl during the dry period (May, 2012), whereas during the wet period (November, 2012), it ranged between 0.61 and 4.96 m bgl (CGWB, 2012). The quality of water in these aquifers is, in general, fresh with sporadic occurrences of higher concentrations of iron and nitrate. There is an occurrence of arsenic contamination at places near-surface aquifers along the course of river Ganga in the extreme north-western, and north-central part of the area. The underlying deeper aquifer is arsenic-free. Besides these, the palaeochannels (Fig. 1a) are localized reservoir of fresh groundwater, and form the near-surface aquifers.

S2: Table S1 Two dimensional contingency chart (frequency)

WT (j)	R (i)					Total
	1	2	3	…….	u	
1	f_{11}	f_{12}	f_{13}	…….	f_{1u}	f_1
2	f_{21}	f_{22}	f_{23}	…….	f_{2u}	f_2
3	f_{31}	f_{32}	f_{33}	…….	f_{3u}	f_3
.	.	.	.	…….	.	.
.	.	.	.	…….	.	.
.	.	.	.	…….	.	.
v	f_{v1}	f_{v2}	f_{v3}	…….	f_{vu}	$f_{v.}$
Total	$f_{.1}$	$f_{.2}$	$f_{.3}$	…….	$f_{.u}$	f_x or f_y

S3: Table S2. Season wise normal rainfall in a morphodynamic system in a part of the Ganga basin of Northern India

**Season**	**Period**	**Rainfall (mm)**	**Percentage (%)**
Winter	October and February	59.1	6.8
Summer	March to May	61.4	7.0
Southwest monsoon	June to September	752.3	86.2
	**Total**	872.8	100.0

S4: Table S3. Detailed well inventory in a morphodynamic groundwater system in a part of the Ganga basin, Northern India

**Well Id.**	**Place (s)**	**X**	**Y**	**Geomorphology**	**RD**	**MP**	**DOW**	**Well Id.**	**Place (s)**	**X**	**Y**	**Geomorphology**	**RD**	**MP**	**DOW**
**W-1**	Chhiyattar	84.890	25.665	Flood Plain	49.50	0.44	6.20	**W-26**	Parsa bazar	85.120	25.551	Flood Plain	49.80	0.62	10.40
**W-2**	Maner	84.890	25.648	Flood Plain	49.40	0.54	7.00	**W-27**	Khazpura	85.080	25.604	Natural Levees	48.00	0.35	12.00
**W-3**	Gyaspur(Purvatola)	84.890	25.627	Flood Plain	50.70	0.65	7.92	**W-28**	Phulwari	85.080	25.578	Natural Levees	48.70	0.80	10.00
**W-4**	Korhar	84.870	25.595	Natural Levees	53.20	0.60	9.00	**W-29**	Dalluchak	85.050	25.586	Back Swamp	49.10	0.32	6.50
**W-5**	Bhishambhrapur	84.890	25.578	Flood Plain	55.60	0.70	9.26	**W-30**	Chheditola	85.040	25.565	Flood Plain	46.80	0.54	5.65
**W-6**	Doghra	84.900	25.592	Flood Plain	54.80	0.60	8.30	**W-31**	Hulastabad	85.030	25.538	Flood Plain	46.90	0.70	6.16
**W-7**	Nagwa	84.910	25.621	Natural Levees	50.70	0.30	9.50	**W-32**	Khadika	85.070	25.528	Flood Plain	48.40	0.62	10.60
**W-8**	Mehnawa	84.910	25.648	Active Flood Plain	49.40	0.66	9.85	**W-33**	Nisirpura	85.090	25.530	Flood Plain	48.70	0.37	12.80
**W-9**	Darbeshpur	84.940	25.652	Active Flood Plain	50.30	0.35	12.70	**W-34**	Saristabad	85.010	25.513	Palaeochannel	45.90	0.50	9.70
**W-10**	Bhateri	84.950	25.634	Back Swamp	51.00	0.75	9.25	**W-35**	Chiraura	84.990	25.521	Palaeochannel	55.50	0.37	7.50
**W-11**	Lalbegwan	84.980	25.651	Back Swamp	50.90	0.62	11.00	**W-36**	Faridpur	84.980	25.549	Flood Plain	53.20	0.70	7.35
**W-12**	Khaspur	85.010	25.637	Natural Levees	50.20	0.50	12.00	**W-37**	Raunia	84.950	25.550	Palaeochannel	52.20	0.65	10.80
**W-13**	Gopalapur	84.940	25.613	Flood Plain	50.90	0.25	8.25	**W-38**	Gonwa	84.920	25.552	Flood Plain	52.60	0.45	8.60
**W-14**	Mahmadpur	84.940	25.584	Flood Plain	51.50	0.55	7.00	**W-39**	Jinpura	84.890	25.547	Palaeochannel	54.80	0.50	8.05
**W-15**	Anisabad	84.990	25.587	Natural Levees	50.90	0.50	5.82	**W-40**	Raghopur	84.860	25.548	Flood Plain	56.80	0.75	6.90
**W-16**	Murarchak	85.020	25.611	Flood Plain	49.70	0.60	11.00	**W-41**	Amraha	84.860	25.529	Palaeochannel	57.30	0.43	6.40
**W-17**	Goptal,Danapur	85.050	25.634	Natural Levees	49.70	0.50	11.50	**W-42**	Tishkhora	84.900	25.527	Flood Plain	52.70	0.35	12.00
**W-18**	Nasriganj-Patna	85.060	25.640	Natural Levees	50.40	0.56	8.00	**W-43**	Azwa	84.950	25.527	Flood Plain	52.90	0.50	6.90
**W-19**	Digha	85.090	25.643	Natural Levees	50.60	0.62	14.00	**W-44**	Snehitola	84.960	25.505	Palaeochannel	54.50	0.40	7.30
**W-20**	Rajapur	85.110	25.633	Natural Levees	49.10	0.50	9.50	**W-45**	Sarsat	84.920	25.509	Palaeochannel	51.70	0.52	9.20
**W-21**	Patna Law College	85.150	25.619	Natural Levees	49.00	0.90	9.50	**W-46**	Darirpur	84.930	25.480	Palaeochannel	59.80	0.50	6.05
**W-22**	Nandla Chapra	85.170	25.586	Back Swamp	50.80	0.55	9.50	**W-47**	Moriyawa	84.860	25.492	Flood Plain	62.40	0.40	4.25
**W-23**	Sornapur	85.150	25.583	Natural Levees	51.00	0.50	10.00	**W-48**	Din Bigha	84.860	25.458	Flood Plain	61.60	0.55	7.26
**W-24**	Mithapur	85.130	25.602	Palaeochannel	46.90	0.78	6.00	**W-49**	Andhra chowki	84.900	25.466	Flood Plain	59.60	0.63	6.14

**W-25**	Etwarpur	85.130	25.575	Back Swamp	50.20	0.40	12.00	**W-50**	Noniatola	84.870	25.438	Palaeochannel	59.60	0.65	10.80

X: Longitude (in degree), Y: Latitude (in degree), RD: Reduced Level (m, amsl), MP: Measuring Point (m), and DOW: Depth of well (m, bgl)

[Detailed well inventory of 50 open wells are presented in **Table S3.** The open wells were rectangular and circular structures, with the depth ranging from 4.25 to 14.00 m below ground level (bgl), with an average of 8.87m. The depth to measuring point (MP) ranged from 0.25 to 0.90 m. Reduce level varied from 45.90 to 62.40 m, amsl, with a mean of 51.92 m, amsl. Groundwater was mainly extracted through the bucket and pulley method for domestic and gardening purposes. Monthly water levels were monitored during the first week of every month from February 2012 to March 2014 from these open wells, which are uniformly spread in the study area (**Fig.1c**). The study of water level fluctuation helps determine the time-wise depth to water level, recharge and discharge periods, hydraulic gradient, and rate of water level increase or decrease (Mondal and Singh, 2004)].

S5: Figure S3. Water level contours map of May 2012, and its fluctuation

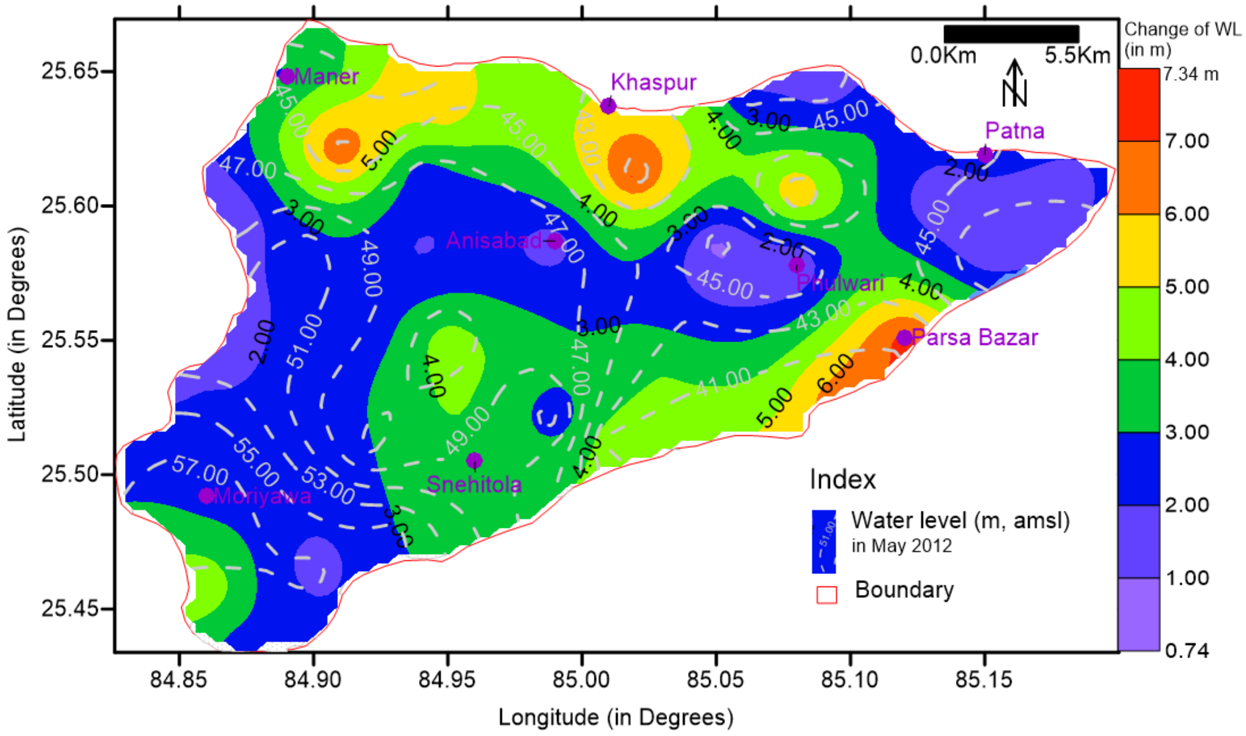

Figure S3. Water level contours map of May 2012, and water level fluctuation due to the monsoon rainfall (N.C.M.: sketched this figure with the help of Surfer ver. 13.4.553, www.goldensoftware.com)

[Groundwater level varied from 39.01 to 59.10 m, amsl, with an average of 47.04 m, amsl during May 2012, whereas it was varied from 44.57 to 61.63 m, amsl, with an average of 50.35m, amsl during October 2012. The water level raised an average of 3.31m. There was no decline in groundwater level after the monsoon, but the groundwater level raised more than 4.00 after the monsoon in the central northern and southern parts of the study area. This exhibits regional groundwater flow direction but not the micro-level characteristics of the aquifer].

S6: Table S4. Estimated percentage natural recharge (%) using information-based model for the monsoon data at the shallow well areas at a morphodynamic system in a part of the Ganga basin

**Well Id.**	**Geomorphology**	Information-based natural groundwater recharge (%) for the monsoon data				
		H(R)	H(WT)	H(R,WT)	T(R,WT)	Re(%)
W-1	Flood Plain	1.906	1.061	2.500	0.467	24.50
W-2	Flood Plain	1.906	1.406	2.500	0.811	42.57
W-3	Flood Plain	1.906	1.299	2.500	0.704	36.97
W-4	Natural Levees	1.906	0.544	2.156	0.294	15.41
W-5	Flood Plain	1.906	0.954	2.250	0.610	32.01
W-6	Flood Plain	1.906	1.299	2.500	0.704	36.97
W-7	Natural Levees	1.906	1.500	2.500	0.906	47.52
W-8	Active Flood Plain	1.906	1.750	2.750	0.906	47.52
W-9	Active Flood Plain	1.906	1.750	2.500	1.156	60.64
W-10	Back Swamp	1.906	1.906	2.750	1.061	55.69
W-11	Back Swamp	1.906	1.906	3.000	0.811	42.57
W-12	Natural Levees	1.906	2.250	3.000	1.156	60.64
W-13	Flood Plain	1.906	1.299	2.500	0.704	36.97
W-14	Flood Plain	1.906	0.954	2.500	0.360	18.90
W-15	Natural Levees	1.906	0.544	2.156	0.294	15.41
W-16	Flood Plain	1.906	2.156	3.000	1.061	55.69
W-17	Natural Levees	1.906	0.954	2.250	0.610	32.01
W-18	Natural Levees	1.906	0.811	2.500	0.217	11.38
W-19	Natural Levees	1.906	0.544	2.156	0.294	15.41
W-20	Natural Levees	1.906	1.299	2.156	1.049	55.04
W-21	Natural Levees	1.906	1.061	2.406	0.561	29.45
W-22	Back Swamp	1.906	1.406	2.500	0.811	42.57
W-23	Natural Levees	1.906	0.544	2.156	0.294	15.41
W-24	Palaeochannel	1.906	0.544	2.156	0.294	15.41
W-25	Back Swamp	1.906	1.406	2.500	0.811	42.57
W-26	Flood Plain	1.906	1.299	2.500	0.704	36.97
W-27	Natural Levees	1.906	2.250	2.750	1.406	73.76
W-28	Natural Levees	1.906	0.000	1.906	0.000	0.00
W-29	Back Swamp	1.906	0.000	1.906	0.000	0.00
W-30	Flood Plain	1.906	1.406	2.500	0.811	42.57
W-31	Flood Plain	1.906	1.406	2.500	0.811	42.57
W-32	Flood Plain	1.906	1.299	2.500	0.704	36.97
W-33	Flood Plain	1.906	1.299	2.500	0.704	36.97
W-34	Palaeochannel	1.906	1.906	2.750	1.061	55.69

W-35	Palaeochannel	1.906	1.299	2.750	0.454	23.85
W-36	Flood Plain	1.906	1.061	2.156	0.811	42.57
W-37	Palaeochannel	1.906	1.750	2.750	0.906	47.52
W-38	Flood Plain	1.906	1.299	2.750	0.454	23.85
W-39	Palaeochannel	1.906	1.299	2.500	0.704	36.97
W-40	Flood Plain	1.906	0.000	1.906	0.000	0.00
W-41	Palaeochannel	1.906	0.544	2.156	0.294	15.41
W-42	Flood Plain	1.906	0.954	2.500	0.360	18.90
W-43	Flood Plain	1.906	1.299	2.750	0.454	23.85
W-44	Palaeochannel	1.906	1.906	3.000	0.811	42.57
W-45	Palaeochannel	1.906	0.544	2.156	0.294	15.41
W-46	Palaeochannel	1.906	1.061	2.500	0.467	24.50
W-47	Flood Plain	1.906	0.811	2.500	0.217	11.38
W-48	Flood Plain	1.906	1.299	2.500	0.704	36.97
W-49	Flood Plain	1.906	0.811	2.500	0.217	11.38
W-50	Palaeochannel	1.906	1.406	2.500	0.811	42.57

S7: Table S5. Estimated the monsoon groundwater reserves

**Well Id**	Geomorphological unit	**Specific yield**	**Soil type**	**Area influenced by well (km^2)**	**MGWR (in 2012)**	**MGWR (in 2013)**
W-1	Flood Plain	0.08	Motichak sandy loam	3.23	0.84	1.46
W-2	Flood Plain	0.08	Motichak sandy loam	3.30	0.79	0.96
W-3	Flood Plain	0.07	Khanpur clay loam	8.97	2.71	2.55
W-4	Natural Levees	0.10	Faijullah sandy clay loam	7.34	0.73	0.29
W-5	**Flood Plain**	0.07	Khanpur clay loam	8.32	1.47	1.48
W-6	Flood Plain	0.07	Khanpur clay loam	9.23	1.93	2.44
W-7	Natural Levees	0.07	Khanpur clay loam	7.23	3.54	3.10
W-8	Active Flood Plain	0.07	Khanpur clay loam	5.84	1.62	1.95
W-9	Active Flood Plain	0.08	Motichak sandy loam	9.00	4.06	4.66
W-10	Back Swamp	0.07	Khanpur clay loam	11.14	4.27	4.75
W-11	Back Swamp	0.07	Khanpur clay loam	9.26	2.65	2.89
W-12	Natural Levees	0.07	Khanpur clay loam	8.74	3.24	3.37
W-13	Flood Plain	0.07	Khanpur clay loam	12.00	2.11	2.68
W-14	Flood Plain	0.07	Khanpur clay loam	15.28	2.00	2.19
W-15	Natural Levees	0.07	Khanpur clay loam	20.30	2.17	2.06
W-16	Flood Plain	0.07	Khanpur clay loam	12.30	**6.26**	**7.61**
W-17	Natural Levees	0.07	Khanpur clay loam	8.24	2.31	2.10
W-18	Natural Levees	0.07	Khanpur clay loam	4.73	0.69	0.76
W-19	Natural Levees	0.07	Khanpur clay loam	7.81	0.77	0.86
W-20	Natural Levees	0.07	Khanpur clay loam	8.35	1.68	1.20
W-21	Natural Levees	0.07	Khanpur clay loam	7.87	1.38	1.42
W-22	Back Swamp	0.07	Khanpur clay loam	11.96	1.38	3.33
W-23	Natural Levees	0.07	Khanpur clay loam	6.87	0.57	0.92
W-24	Paleochennel	0.07	Khanpur clay loam	11.75	1.09	1.18
W-25	Back Swamp	0.07	Khanpur clay loam	9.75	2.27	2.48
W-26	Flood Plain	0.07	Khanpur clay loam	8.58	4.41	4.67
W-27	Natural Levees	0.07	Khanpur clay loam	14.59	6.16	4.11
W-28	Natural Levees	0.07	Khanpur clay loam	14.23	0.93	0.78
W-29	Back Swamp	0.07	Khanpur clay loam	12.18	0.63	0.84
W-30	Flood Plain	0.07	Khanpur clay loam	15.02	2.37	1.82
W-31	Flood Plain	0.07	Khanpur clay loam	16.16	4.50	3.18

W-32	Flood Plain	0.07	Khanpur clay loam	12.24	3.92	3.56
W-33	Flood Plain	0.07	Khanpur clay loam	7.38	3.21	3.03
W-34	Paleochennel	0.07	Khanpur clay loam	8.73	2.71	3.21
W-35	Paleochennel	0.07	Khanpur clay loam	11.72	2.01	1.24
W-36	Flood Plain	0.07	Khanpur clay loam	15.07	3.78	2.00
W-37	Paleochennel	0.07	Khanpur clay loam	10.33	3.26	2.68
W-38	Flood Plain	0.07	Khanpur clay loam	12.35	2.10	2.47
W-39	Paleochennel	0.07	Khanpur clay loam	9.12	1.44	1.58
W-40	Flood Plain	0.10	Faijullah sandy clay loam	**3.19**	**0.38**	**0.04**
W-41	Paleochennel	0.10	Faijullah sandy clay loam	11.63	2.45	2.36
W-42	Flood Plain	0.08	Rasaibigha clay loam	12.00	2.42	2.03
W-43	Flood Plain	0.08	Rasaibigha clay loam	9.83	3.21	2.62
W-44	Paleochennel	0.08	Rasaibigha clay loam	14.00	4.50	4.91
W-45	Paleochennel	0.08	Rasaibigha clay loam	12.66	2.95	1.03
W-46	Paleochennel	0.08	Rasaibigha clay loam	11.52	2.76	2.30
W-47	Flood Plain	0.08	Rasaibigha clay loam	**20.12**	4.07	5.12
W-48	Flood Plain	0.08	Rasaibigha clay loam	8.10	3.18	3.14
W-49	Flood Plain	0.08	Rasaibigha clay loam	16.21	2.04	2.79
W-50	Paleochennel	0.08	Rasaibigha clay loam	5.21	1.13	1.21
			**Minimum**	**3.19**	**6.26**	**7.61**
			**Maximum**	**20.30**	**0.38**	**0.04**
			**Average**	**10.42**	**2.46**	**2.43**
			**Total**	**521.00**	**123.03**	**121.38**

S8: Figure S4

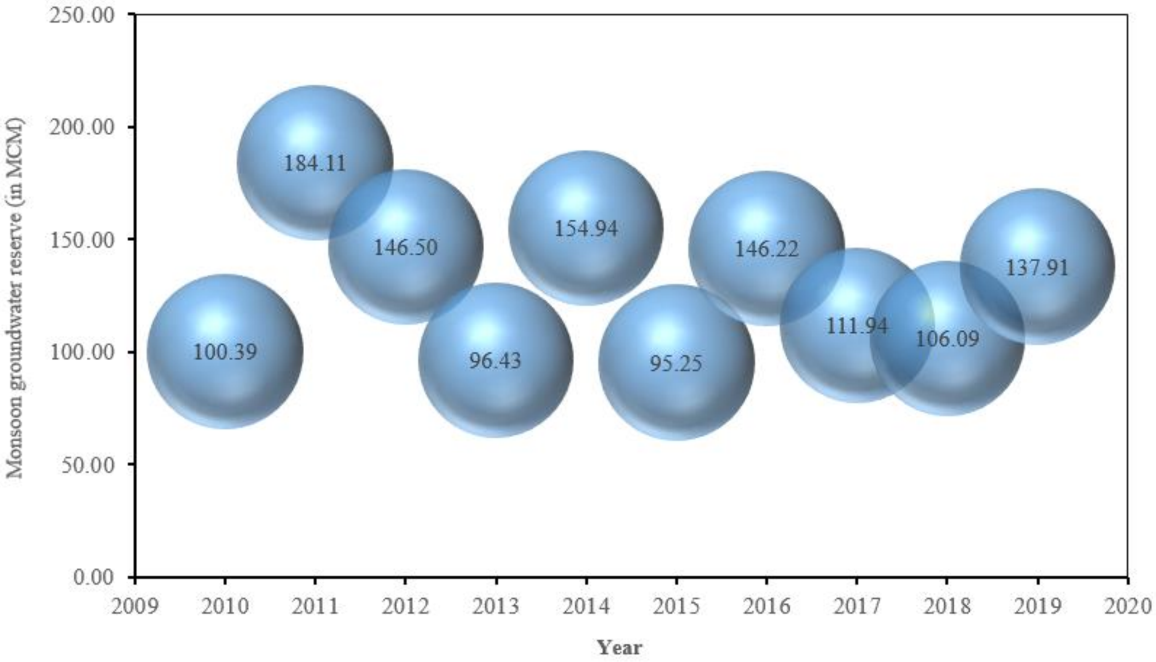

Figure S4. Natural groundwater reserve in a morphodynamic system at a part of the Ganga basin of Northern India in the period of 2010-2019

Central Ground Water Board (CGWB). *Background Report on Pilot Micro Level Aquifer Mapping Projects, AQBHR area, Patna, Bihar, p.95* (2012).

Mondal et al. *Heliborne Transient Electromagnetic Investigation in Middle Ganga Plains, Parts of Patna District, Bihar.* Restricted Technical Report No.: NGRI-2015-GW-868, January 2015, p.48 (2015).

Mondal et al. *Mid Term Report, AQBHR Area, Patna, Bihar under the AQUIM Project*. Aquifer Characterization using Advance Geophysical Techniques in Representative Geological Terrains of India, Technical Report No. NGRI-2014-GW-856, July 2014, p. 52 (2014).

Mondal, N.C., Singh, V.S. A new approach to delineate the groundwater recharge zone in hard rock terrain. *Curr Sci India*, **87**, 5, 658-662 (2004).
